# A case report of HIV-1 superinfection in an HIV controller leading to loss of viremia control: a retrospective of 10 years of follow-up

**DOI:** 10.1186/s12879-019-4229-3

**Published:** 2019-07-05

**Authors:** Diogo Gama Caetano, Fernanda Heloise Côrtes, Gonzalo Bello, Suwellen Sardinha Dias de Azevedo, Brenda Hoagland, Larissa Melo Villela, Beatriz Grinsztejn, Valdiléa Gonçalves Veloso, Monick Lindenmeyer Guimarães, Mariza Gonçalves Morgado

**Affiliations:** 1 Laboratório de Aids e Imunologia Molecular, Instituto Oswaldo Cruz (IOC) –FIOCRUZ, Av. Brasil 4365, Rio de Janeiro, RJ 21045-900 Brazil; 2 Instituto Nacional de Infectologia Evandro Chagas (INI), Laboratório de Pesquisa clínica em DST e Aids, Rio de Janeiro, Brazil

**Keywords:** HIV-1, HIV-controllers, LTNP, Superinfection, Dual infection

## Abstract

**Background:**

HIV controllers (HICs) are a rare group of HIV-1-infected individuals able to naturally control viral replication. Several studies have identified the occurrence of HIV dual infections in seropositive individuals leading to disease progression. In HICs, however, dual infections with divergent outcomes in pathogenesis have been described.

**Case presentation:**

Here, we present a case report of a HIC diagnosed in late 1999 who displayed stable CD4^+^ T cell levels and low plasmatic viral load across 12 years of follow-up. In early 2013, the patient started to present an increase in viral load, reaching a peak of 10,000 copies/ml in early 2014, followed by an oscillation of viremia at moderate levels in the following years. The genetic diversity of *env* proviral quasispecies from peripheral blood mononuclear cells (PBMCs) was studied by single genome amplification (SGA) at six timepoints across 2009–2017. Phylogenetic analyses of *env* sequences from 2009 and 2010 samples showed the presence of a single subtype B variant (called B_1_). Analyses of sequences from 2011 and after revealed an additional subtype B variant (called B_2_) and a subsequent dominance shift in the proviral quasispecies frequencies, with the B_2_ variant becoming the most frequent from 2014 onwards. Latent syphilis related to unprotected sexual intercourse was diagnosed a year before the first detection of B_2,_ evidencing risk behavior and supporting the superinfection hypothesis. Immunologic analyses revealed an increase in CD8^+^ and CD4^+^ T cell immune activation following viremia increase and minor T cell subset alterations during follow-up. HIV-specific T cell responses remained low throughout the follow-up period.

**Conclusions:**

Altogether, these results show that loss of viremia control in the HIC was associated with superinfection. These data alert to the negative consequences of reinfection on HIV pathogenesis, even in patients with a long history of viremia control and an absence of disease progression, reinforcing the need for continued use of adequate prevention strategies.

## Background

HIV controllers (HICs) are a rare group of HIV-1-infected individuals able to naturally control viral replication. A fraction of those individuals is also classified as long term nonprogressors (LTNP), as they maintain CD4^+^ T cell counts > 500 cells/mm^3^ during more than 10 years of infection without progressing to AIDS in the absence of antiretroviral treatment [1].

An aspect of HIV infection is the possibility of infections by two or more phylogenetically distinct and unrelated variants in a single individual, characterizing a dual infection (DI). More specifically, these DIs are classified as coinfections when all variants are concomitantly acquired in a single transmission event or superinfections (SIs) when the viruses originate from multiple subsequent transmission events [2]. HIV DI has been described in several studies with significant prevalence mainly among key populations [3–7] and is related to a faster progression to AIDS [8–10]. DIs were also observed among LTNPs and/or HICs [11–15], with variable consequences on HIV pathogenesis. While some individuals retain spontaneous disease control [11–13], others present loss of viremia control and experience disease progression [11, 14, 15].

Here, we report the case of an HIV-1 positive individual with natural control of viral replication and no progression to AIDS over more than 10 years of clinical follow-up who presented a partial loss of viremia control after an SI event.

## Case presentation

Subject VC06 is a 40-year-old, transgender woman from Rio de Janeiro, Brazil, who was diagnosed with HIV-1 infection at the end of 1999 and has been seen for routine clinical follow-up at the Instituto Nacional de Infectologia Evandro Chagas (INI-Fiocruz), Rio de Janeiro, Brazil since 2005. In 2009, VC06 signed an informed consent and was enrolled in the INI-Fiocruz LTNP/HIC cohort study, approved by the Brazilian National Human Research Ethics Committee (CONEP 840/2008) and by the FIOCRUZ *Research Ethics* Committee (CEP 1717.0.000.009–07). Due to study enrollment, individual VC06 was followed at least once every 6–12 months to perform specific infection-monitoring tests (such as HIV-1 RNA viral load quantification and CD4^+^ T cell counts) and routine clinical laboratory exams and to assess data related to clinical status and exposure to sexually transmitted infections. In addition, blood was collected at each visit to isolate plasma, whole blood and peripheral blood mononuclear cell (PBMC) samples for study. Subject VC06 was initially classified as an LTNP HIV viremic controller (< 2000 cp/ml dually infected with two HIV-1 subtype B viruses (de Azevedo et al. 2017) [16]. She carries a nonprotective HLA-B genotype (HLA-B*15:01/ B*48:02) but has heterozygosis for the CCR5-Δ32 mutation, which is considered a host-protective allele for disease infection and progression.

**Fig. 1:**
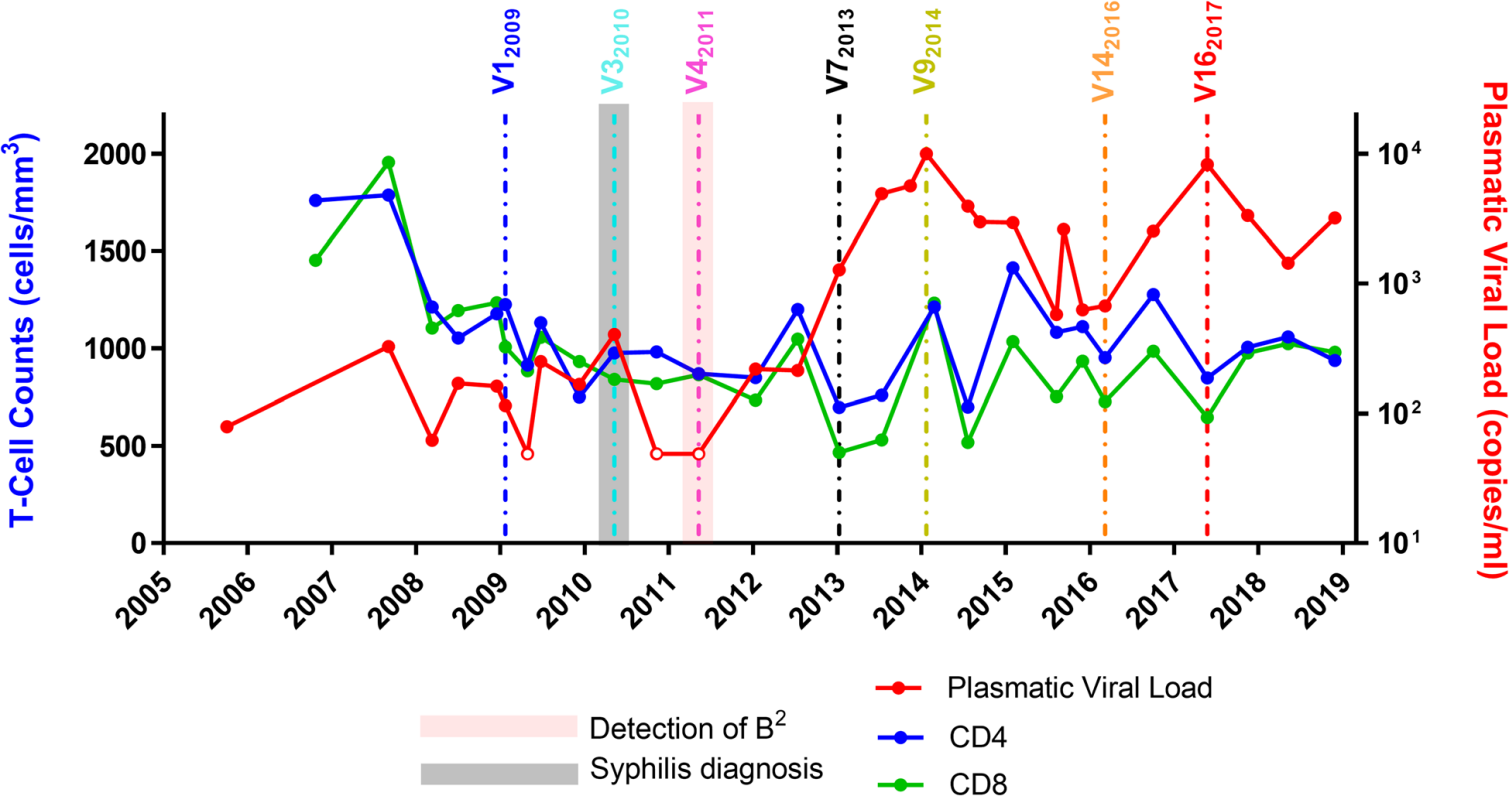
T cell counts (cells/mm^3^) and plasmatic viral load (copies/ml) of VC06. The first syphilis diagnosis point and the first point of detection of B_2_ are indicated by shaded areas, while the visits used for experiments are indicated by dashed lines in the graph. Time points with plasmatic viral load < 50 copies/ml are indicated by open circles

Subject VC06 displayed low-level viremia (< 500 copies/mL) in the absence of antiretroviral therapy until early 2013, when she started to show increases in the viral load, reaching approximately 10,000 copies/ml 1 year later (Fig. 1, V9_2014_). The following months were associated with a spontaneous decrease in viral load, reaching 577 copies/ml in August 2015. Combination antiretroviral therapy (cART) with a scheme containing TDF, 3TC, and EFZ was prescribed in November 2015 but interrupted 1 month later by the patient due to intense dizziness related to the treatment. Side effects ceased, but the continuity of the therapy was refused by the patient in the following years. Transient recovery of viremia control was followed by intermittent viral loads above 2000 copies/ml and a new peak of approximately 8000 copies/ml in May 2017. This new peak of viremia was followed by a spontaneous decrease in viral load, reaching 1435 copies/ml in May 2018. The most recent available data indicated a viral load of approximately 3500 copies/ml at the end of 2018 (Fig. 1). Despite increasing viremia, CD4^+^ T cell counts during the whole period were stable at high levels, suggesting no immunological commitment or disease progression.

In addition to the intermittence of the plasmatic viral load after a controller period, individual VC06 was diagnosed with latent syphilis in May 2010 based on positive VDRL results (1/32) in the absence of clinical signs or symptoms and a previous negative VDRL test in December 2009. The syphilis diagnosis coincided with unprotected sexual intercourse reported by the patient, and treatment with weekly benzathine benzylpenicillin 1,200,000 IU intramuscular injections were administered for 3 weeks starting in November 2010. Late latent syphilis was further diagnosed again at two additional timepoints: first in September 2015, based on VDRL titer of 1/8; second in November 2017, based on TPHA positive and a VDRL titer of 1/512. Both cases were preceded by a VDRL titer of 1/1 6 months before and were treated, as described above, in November 2015 and May 2018, respectively. No clinical signs or symptoms associated with syphilis infection were observed during follow-up. Another clinical event during the follow-up period included the diagnosis of an anal fistula at the beginning of 2014, which was surgically treated in the same year.

**Fig. 2:**
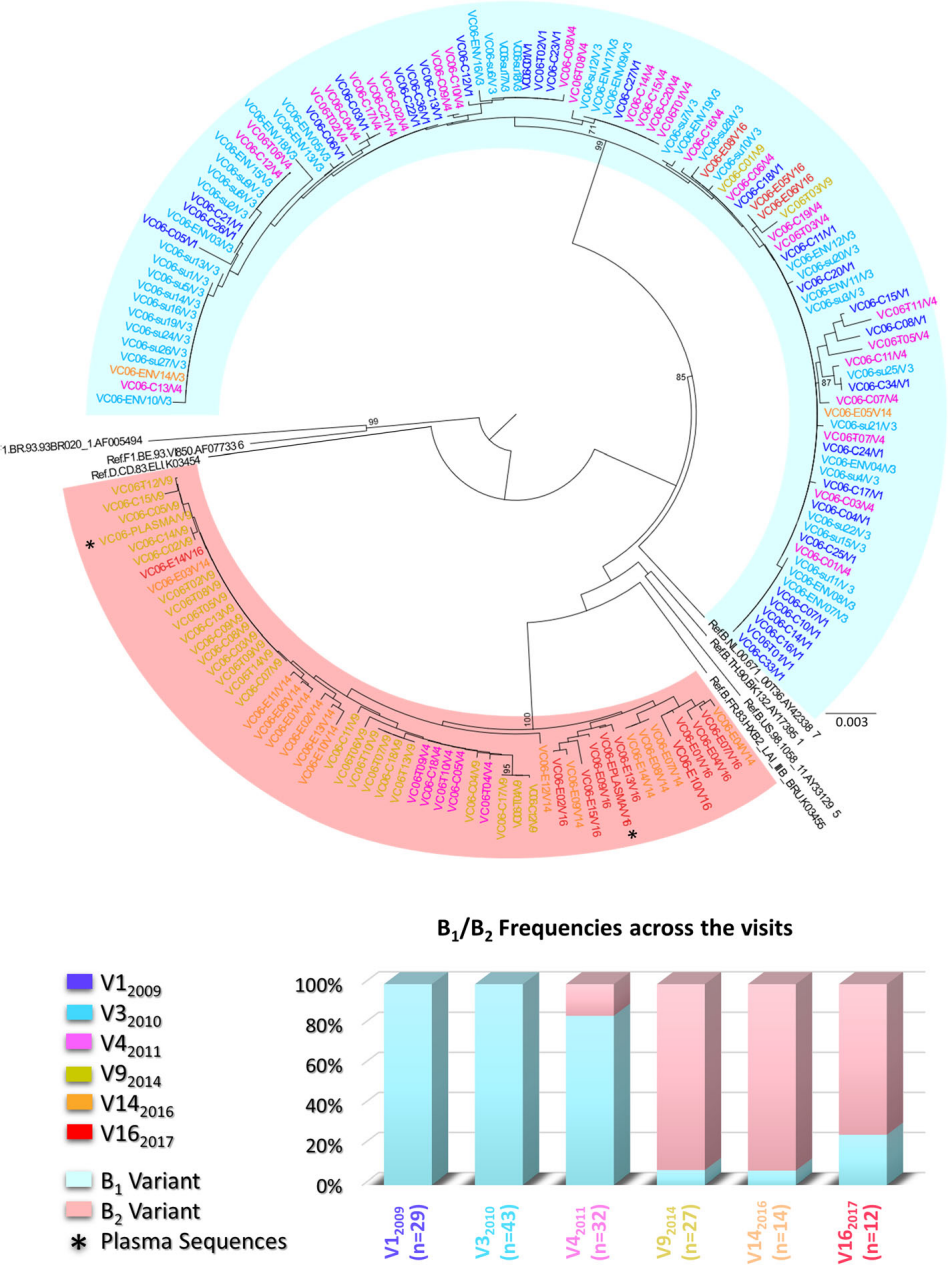
Longitudinal analysis of proviral and plasma HIV-1 *env* sequences obtained from VC06 during 2009–2017. Sequence names are colored according to their respective visit. Clusters containing B_1_ and B_2_ sequences are shaded in blue and red, respectively. For each visit, the total number of sequences and proportion of B_1_ and B_2_ variants are indicated on the stacked bar graph and colored according to the legend. The tree was constructed using the Tamura Nei model and 1000 bootstrap replicates in MEGA 6 software. Bootstrap values lower than 75% are not shown. Reference sequences for HIV-1 subtypes D, F1, and B were used as an outgroup and are shown in black

To assess the patterns of intrahost viral evolution and to investigate the cause associated with the loss of viremia control, PBMCs (1 × 10^7^ cells) from selected visits (Fig. 1) were thawed and used for genomic DNA extraction, as previously described [17]. The genomic DNA obtained was used for amplification by nested PCR single genome amplification (SGA) and sequencing of a ≈ 600 bp C2-C4 fragment of HIV-1 *env*, as previously described [18]. A neighbor-joining phylogenetic tree containing all sequences obtained from samples collected over time is shown in Fig. 2. *Env* sequences from 2009 (V1_2009_; *n* = 29) and 2010 (V3_2010_; *n* = 43) samples showed the presence of a single subtype B variant (called B_1_). Analysis of *env* sequences from 2011 (V4_2011_; *n* = 32), 1 year after the diagnosis of syphilis infection, showed the presence of a second subtype B variant (called B_2_) in addition to the previous B_1_ variant. These variants branched separately and displayed a mean *env* genetic distance of 16.8%. Tropism analyses, realized through Geno2pheno tool using a false-positive rate (FPR) of 10% [19], of *env* sequences, obtained at all time points, showed that both B_1_ and B_2_ variants correspond to R5-tropic viruses that present different predominant motifs at the top of the V3 loop (QPGR/QPGG for B_1_ and GPGR for B_2_). *Env* analyses of samples from subsequent time points revealed a shift in the proviral quasispecies proportion, with an increase of B_2_ variant frequency from 16% in 2011 (V4_2011_; *n* = 32) to 93% in 2014 (V9_2014_; *n* = 27). The majority of the B_2_ (93%) viral quasispecies was maintained even after the reduction of plasmatic viral load in 2016 (V14_2016_; *n* = 14) as well as after a new peak of viremia (75%) in 2017 (V16_2017_; *n* = 12) (Fig. 2). For B2 quasispecies from all timepoints (*n* = 52), 80% of the sequences obtained were classified as R5 with FPR values greater than 45%, while the remaining presented FPR values between 11.5% and 18,5%.

Plasma sequences were obtained from the V9_2014_ and V16_2017_ samples, as previously described [18], supporting that B_2_ was the replicating variant accounting for the increase in viremia observed at both time points (Fig. 2). Moreover, the distribution of B_2_ sequences on the *env* phylogenetic tree indicates a pattern of increasing divergence along the follow-up period, with the identification of two distinct clusters harboring most sequences for the V9_2014_ and V16_2017_ samples_._ In additional analyses, all B_2_ sequences were used for estimation and reconstruction of the most recent common ancestor (MRCA), using the Phylip package [20]. The mean genetic distance between the MRCA and the B_2_ sequences for each visit was calculated to determine B_2_ viral divergence during follow-up. We observed increasing values of divergence during follow-up (0.9% for V4_2011_, 1.7% for V9_2014_; 3.5% for V14_2016_; 4.4% for V16_2017_), indicating a temporal evolution pattern. These results, in addition to the observation that sequences from the V14_2016_ samples were distributed between the V9_2014_ and V16_2017_ clusters, with no clear predominance of any population, also indicate that different B2-related viral quasispecies accounted for the viral replication in each viremia peak.

**Fig. 3:**
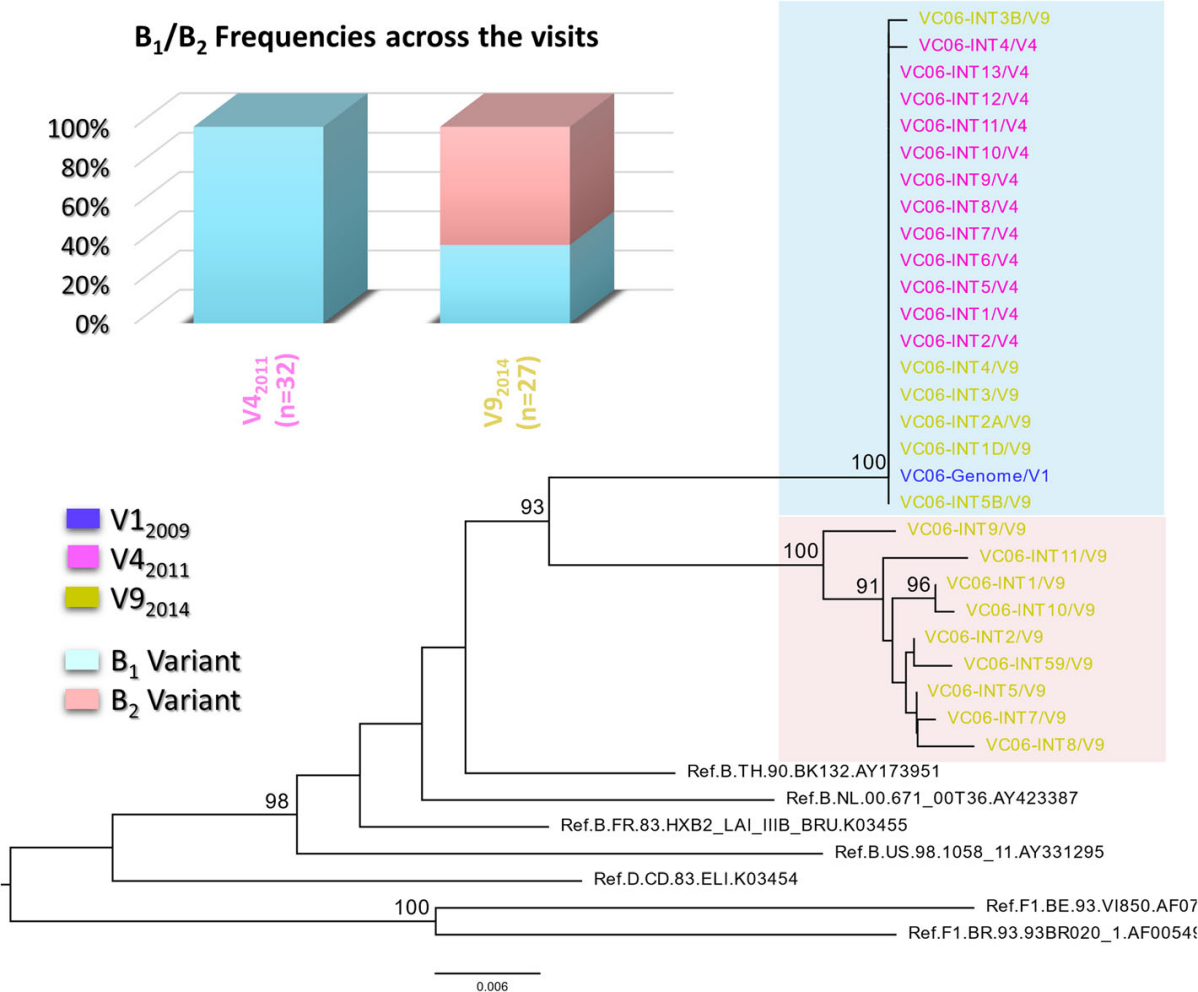
Longitudinal analysis of proviral HIV-1 integrase sequences obtained from VC06 during 2011 and 2014. Sequence names are colored according to their respective visit, clusters containing B_1_ and B_2_ sequences are shaded in blue and red, respectively, and the total number of sequences and proportion of B_1_ and B_2_ variants are indicated on the stacked bar graph, as previously described. *Int* sequence obtained from B_1_ full length genome is included to confirm B_1_ cluster identity. The tree was constructed using the Tamura Nei model and 1000 bootstrap replicates in MEGA 6 software. Bootstrap values lower than 75% are not shown. Reference sequences for HIV-1 subtypes D, F1, and B were used as an outgroup and are shown in black

To better characterize the SI and assess the divergence between B_1_ and B_2_ at a more conserved region of the viral genome, we conducted SGA of the *int* region from the V4_2011_ sample, in which we first detected the B_2_ variant, and from the V9_2014_ sample, in which B_2_ became the dominant variant. A neighbor-joining phylogenetic tree containing the *int* sequences is shown in Fig. 3. In the V4_2011_ sample (obtained at the time of SI detection), a single *int* variant related to B_1_was found despite the detection of two variants in the *env* analysis from the same time point. The absence of a second *int* variant is probably related to the low number of sequences obtained at this time point (12 sequences for *int* vs 32 sequences for *env*). In the V9_2014_ sample, however, we identified two *int* variants with a mean genetic distance of 4.2%. Although these data indicate the presence of B_1_ and B_2_, the frequencies of both *int* variants differ from those observed for *env* (40% vs 7% for B_1_; 60% vs 93% for B_2_). Even though different fragments have distinct PCR efficiencies, which could introduce a bias factor, the great divergence of representation could be indicative of recombination between *int* and *env* genes.

Finally, to evaluate viral integrity, we obtained the full-length HIV-1 genome from the V1_2009_ sample, as previously described [21]. The overlapping fragment sequences obtained allowed the identification of the B_1_ variant full-length genome and the absence of deletions or frame-shift alterations related to genetic defects, indicating that B_1_ is a replication-competent virus. The full-length B_1_ genome also confirmed that the *int* variants obtained via SGA of the V4_2011_ sample are related to this variant (Fig. 3). Isolation of the full-length genome for the B_2_ variant was not possible due to the equivalent frequencies of B_1_ and B_2_ in some genes, as demonstrated by *int* SGA, which could lead to the generation of PCR artifacts.

To understand the potential impact of the SI on the host immune system, we analyzed alterations during the follow-up period in the frequencies of the T cell subsets and immune response to HIV peptides in PBMC samples collected at V3_2010_ (prior to SI), V4_2011_ (at the moment of B2 *env* variant identification after SI onset), V7_2013_ (prior to the first peak of viremia), V9_2014_ (at the first peak of viremia and detection of B_1_ and B_2_ *env* and *int* variants), V14_2016_ (after viremia control) and V16_2017_ (at the second peak of viremia). Briefly, T cell activation was evaluated by multiparametric flow cytometry by staining the cells with anti-CD3, anti-CD4 or anti-CD8, anti-CD38 and anti-HLA-DR antibodies to determine the frequencies of CD38^+^HLA-DR^+^ cells in both CD4^+^ and CD8^+^ subsets, as previously described [22]. In addition, cells were also labeled with anti-CD45RA, anti-CD27 and anti-CD95 antibodies to evaluate the frequencies of naïve (TN; CD45RA^+^CD27^+^CD95^−^), system memory (TSCM; CD45RA^+^CD27^+^CD95^+^), central memory (TCM; CD45RA^−^CD27^+^CD95^+^), effector memory (TEM; CD45RA^−^CD27^−^CD95^+^) and effector (TEFF; CD45RA^+^CD27^−^CD95^+^) T cell subsets.

**Fig. 4:**
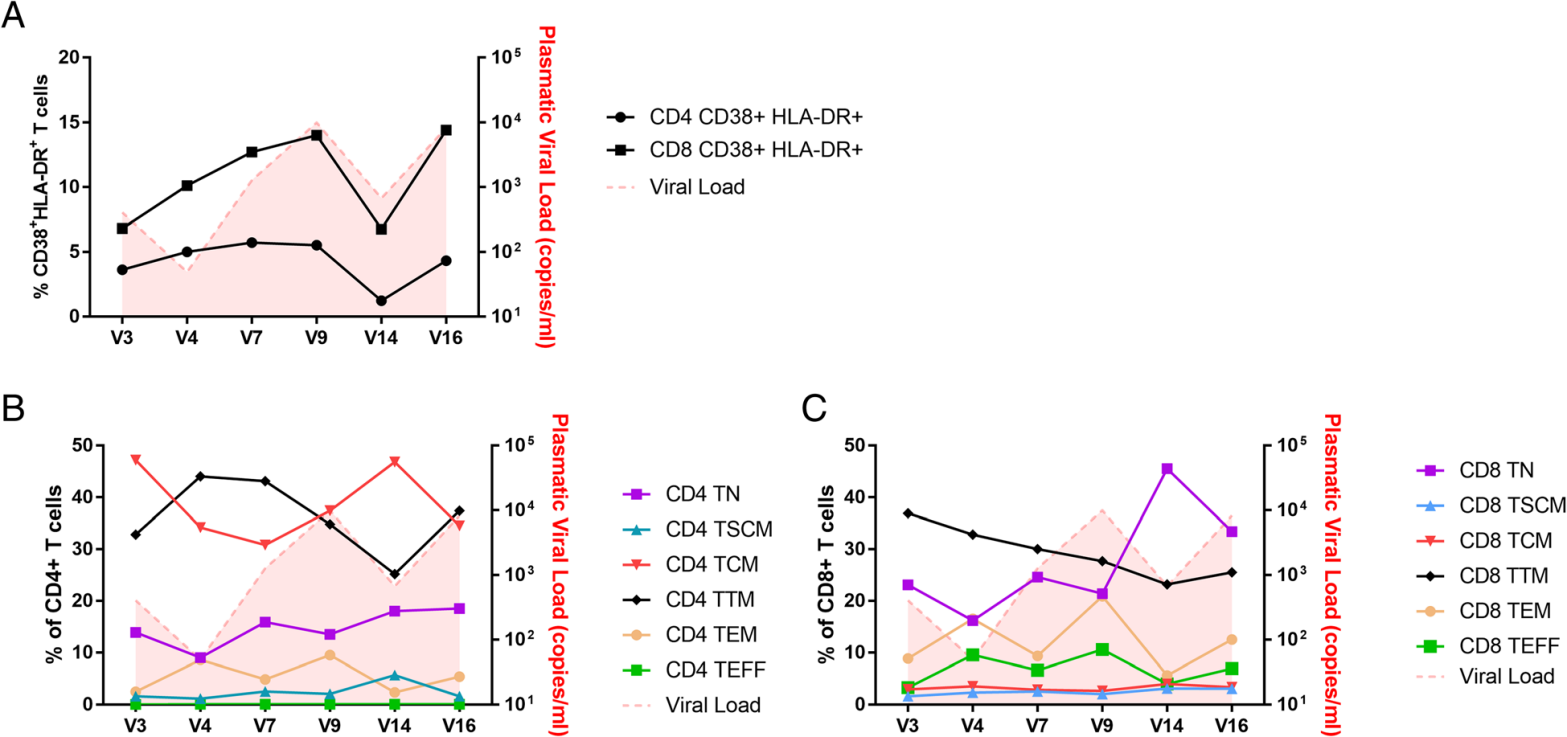
T cell profile of VC06 during follow-up between 2010 and 2017. Frequencies of activated T cells (CD38^+^HLA-DR^+^) are shown in graph (**a**); frequencies of naïve (TN; CD45RA^+^CD27^+^CD95^−^), stem-memory (TSCM; CD45RA^+^CD27^+^CD95^+^), central-memory (TCM; CD45RA^−^CD27^+^CD95^+^), effector-memory (TEM; CD45RA^−^CD27^−^CD95^+^) and effector (TEFF; CD45RA^+^CD27^−^CD95^+^) T cells are shown in graphs (**b**) and (**c**). For all graphs, red shaded areas indicate the plasmatic viral load for each visit analyzed

An increase in the percentage of CD8^+^CD38^+^HLA-DR^+^ T cells was observed in samples from V3_2010_ (6.81%) to V9_2014_ (14%), followed by a decrease at V14_2016_ (6.76%) and a new peak at V16_2017_ (14.40%) (Fig. 4a). This higher values in the V4_2011_ sample than in the V3_2010_ sample, despite the lower plasmatic viral load, could be suggestive of an association between the SI event and an increase in immune activation. After the emergence of B_2_, the percentage of CD8^+^CD38^+^HLA-DR^+^ T cells followed plasmatic viral load levels, highlighting the relationship between the antigen viral load and CD8^+^ T cell activation. Although at more discrete levels, the same trend was also observed for CD4^+^ T cells (Fig. 4a). In relation to the CD4^+^ T cell subsets (Fig. 4b), we observed a decrease in the frequency of TCM cells between the V4_2011_ and V14_2016_ samples, with the recovery of those cells in the V16_2017_ sample and an inverse pattern observed for TTM cells. The frequency of CD4^+^ TEM cells reached the highest levels at visits near the detection of the superinfection (V4_2011_) and at both peaks of viremia (V9_2014_ and V16_2017_). For CD8^+^ subsets (Fig. 4c), the frequency of TEM and TEFF cells followed the viral load dynamics, which was in contrast with the expected TN cells expansion after the first viral load peak. Despite these variations, no statistical correlations between the frequencies of the different T cell subsets and plasmatic viral load were found during the follow-up.

In addition, we used Gag and Nef HIV-1 peptides matching CTL epitopes, based on the VC06 HLA-B genotype, to evaluate the HIV-specific T cell response by IFN-γ ELISpot assay and intracellular cytokine and CD107 staining. In general, very low or undetectable HIV-1-specific responses were observed, with the exception of the V9_2014_ sample, when approximately 1% of CD107^+^CD8^+^ T cells showed detectable Gag- or Nef-specific responses (data not shown). No pattern of increase/decrease in the cytokine response was observed in consequence of the antigenic stimulation related to the viral load peak.

## Discussion and conclusions

Here, we report the case of a transgender HIC who experienced a partial loss of viremia control after HIV-1 intrasubtype SI with another subtype B variant. Through molecular analyses of the HIV-1 *env* gene during clinical follow-up, we were able to trace the SI to some time between mid-2010 and mid-2011. At the time, VC06 already had 10 years of diagnosed HIV-1 infection with consistently low viremia and high counts of CD4^+^ T cells in the absence of antiretroviral therapy. This finding indicates that despite natural protection against disease progression, HICs can still be at risk for subsequent infections with new variants, reinforcing the need for continued use of prevention strategies.

The mechanisms underlying viremia and disease progression control phenotypes are not yet fully understood. Studies have shown that they could be associated with host genetic background [23–25], virological characteristics [26, 27], and low levels of immune activation and preservation of memory T cells, among other immunological aspects [28]. CCR5 genetic analysis of the VC06 patient identified heterozygosity for the Δ32 allele (CCR5wild type/CCR5Δ32), a mutated allele previously associated with protection against infection when presented in homozygosis and that has a partial protective role or delayed AIDS progression in heterozigosis [29–33]. However, the association of this genetic characteristic with the of HIV pathogenesis and superinfection could not be established in the present case study.

Although the existence of X4-tropic variants after the superinfection could explain the loss of viremia control in this context, our tropism analyses identified only R5-tropic quasispecies. Despite phenotypic analyses for tropism characterization were not realized, the genotypic analysis in geno2pheno tool had been previously showed to be highly concordant when compared to in vitro assays [34–37]. Moreover, most of the sequences presented high FPR values and lower FPR values were found only in a few sequences representing minoritary variants that should not have a great impact on disease progression or viremia. In the whole, although we can not rule out completely, it is very unlikely that loss of control in VC06 might be associated with the onset of an X4-tropic virus after superinfection. In addition to the VC06 genetic background described in the present study, the diversity of protective elements described in the literature suggest that the control phenotype is not determined by a single factor but is rather probably a result of a set of host and virus characteristics acting synergistically.

Only a few studies are available in the literature with cases of SI in HICs or LTNPs. For some of those studies, SI was associated with disease progression in HICs soon after reinfection [14, 15, 38, 39], as previously observed for noncontrollers [8–10]. However, other studies showed that HICs are able to maintain high CD4^+^ T cell counts and plasmatic viral load at low or undetectable levels after SI [11–13, 18, 40, 41]. This sustained progression control, however, seems to have some complexity, as some of those individuals maintain stable CD4^+^ T cell counts despite experiencing transient viremia incompatible with a previous clinical history [11–13, 18, 40, 41]. The data presented in this study, along with those previously published, reaffirm the complexity of the control phenotype and show that a previous control profile of a single variant does not guarantee immediate and/or efficient control of subsequent infections.

Despite reinfection at the end of 2010, VC06 only started to present increasing viremia at the beginning of 2013, with a peak of 10,000 copies/ml 1 year later. In some cases, the HIC phenotype is the result of infection with defective or attenuated viral strains [42–44]. Previously, Braibant et al. [14] demonstrated the case of an elite controller previously infected with a defective virus who presented disease progression after SI with a competent HIV-1 variant. In our study, the lack of genetic defects in the B_1_ complete genome sequence indicates that B_1_ is a replication-competent virus, and the partial viremia control and the absence of disease progression after entry of the second variant pointed to an inherent and differential ability of the VC06 immune system to control HIV infection, compatible with the HIC phenotype.

The delay in viremia increase also indicates that the loss of viremia control was related to some evolutionary dynamics of both the B_1_ and B_2_ variants and not only to the entry of a new virus_._ Part of this dynamic could be related to recombination processes occurring between the B_1_ and B_2_ variants. Recombination is an important mechanism of diversity generation and immune escape [45] observed in many cases of SI [14, 38, 46–48], including in some HICs who developed disease progression [14, 38]. The frequency discrepancy for B_1_-related *int* vs *env* sequences via SGA suggested the presence of recombinant B_1_B_2_. Although a PCR bias could also explain this difference, a variation of more than 30% in the representativity between the two fragments is less likely to occur due to PCR efficiency. In addition, the sample dilution prior to PCR, that is characteristic of the SGA methodology, should soften the template competition. Albeit this indicates that the differences observed are really due to a variation at the balance between B_1_ and B_2_ variants in *int* vs *env*, our analyses did not observed a recombination point in *int*.

Another sign of the importance of evolutionary dynamics for the clinical consequences of SI for VC06 was the observation of different B_2_ *env* clusters associated with both the V9_2014_ and V16_2017_ samples plus the increase of viral divergence between 2011 and 2017. These data also support the hypothesis that partial loss of viremia control is related to the escape of specific viral populations from the immune response.

The pattern of increase or decrease in the percentage of CD38^+^HLA-DR^+^ T cells following alterations in the plasmatic viral load also points toward the participation of the immune response in the management of the infection during follow-up. T cell subset analyses showed an increase in the proportion of T cells with an effector phenotype (TEM and TEFF) at the timepoints close to SI and of increased viremia, suggesting a possible role for these cells in controlling viral replication. Although no expressive alterations in the Gag and Nef HIV-specific responses were observed during follow-up, this result did not exclude the presence of an HIV-specific immune response to regions other than those analyzed by us.

Finally, it is important to highlight that VC06 continued to maintain high and stable CD4^+^ T cell counts despite the partial loss of viremia control. These data support that virological and immunological control are not necessarily concomitant. Together with other studies that described superinfected HICs with no alterations in CD4^+^ T cell counts but a loss of viremia control [11–13, 18, 40, 41], our data indicate that moderate viremia, in some cases, is not able to impair immunological control.

Despite the partial loss of viremia control and the policy of the Brazilian Ministry of Health indicating antiretroviral treatment to all HIV-infected individuals, VC06 refused to initiate cART. The knowledge of her HIC status and the maintenance of immunological control together with side effects observed during a short period of cART were motivations for this refusal. This patient has been followed-up every 6 months to assess her immunological, clinical and virological status, and no signal of disease progression has been detected thus far. At all clinical visits, cART has again been offered.

Overall, this case raises awareness of the need for continued use of adequate preventive strategies after HIV-1 infection, even in patients with a long history of viremia control and an absence of disease progression. For HICs, our data demonstrate that natural control of HIV-1 replication can be a labile state since the underlying mechanisms associated with this phenotype do not guarantee unrestricted control of any other variant. More studies identifying the factors associated with control of multiple variants in HICs can be an important pathway to identify factors associated with natural control.

## Data Availability

HIV-1 *env* sequences generated from V4_2011_ sample were previously deposited in GenBank under the accession numbers KY852775-KY852806. The remaining sequences generated during the current study were deposited in GenBank under the accession numbers MK757267-MK757421.

## Abbreviations

cART: Combination antiretroviral therapy
CTL: Cytotoxic T Lymphocyte
DI: Dual-infection
*env*: env gene
HICs: HIV controllers
*int*: integrase gene
LTNP: Long-term non progressor
PBMC: Peripheral blood mononuclear cells
SGA: Single genome amplification
SI: Superinfection
TCM: Central-memory T cell
TEFF: Effector T cell
TEM: Effector-memory T cell
TN: Naive T cell
TPHA: Treponema pallidum hemagglutination assay
TSCM: Stem-memory T cell
VDRL: Venereal disease research laboratory test
